# Degraded Environments Alter Prey Risk Assessment

**DOI:** 10.1002/ece3.388

**Published:** 2013-01-10

**Authors:** Oona M Lönnstedt, Mark I McCormick, Douglas P Chivers

**Affiliations:** 1 ARC Centre of Excellence for Coral Reef Studies and School of Marine and Tropical Biology, James Cook UniversityTownsville, Qld 4811, Australia; 2 Department of Biology, University of SaskatchewanSaskatoon, SK S7N 5E2, Canada

**Keywords:** Chemical alarm cues, coral degradation, coral reefs, *Pomacentrus amboinensis*, predation, *Pseudochromis fuscus*

## Abstract

Elevated water temperatures, a decrease in ocean pH, and an increasing prevalence of severe storms have lead to bleaching and death of the hard corals that underpin coral reef ecosystems. As coral cover declines, fish diversity and abundance declines. How degradation of coral reefs affects behavior of reef inhabitants is unknown. Here, we demonstrate that risk assessment behaviors of prey are severely affected by coral degradation. Juvenile damselfish were exposed to visual and olfactory indicators of predation risk in healthy live, thermally bleached, and dead coral in a series of laboratory and field experiments. While fish still responded to visual cues in all habitats, they did not respond to olfactory indicators of risk in dead coral habitats, likely as a result of alteration or degradation of chemical cues. These cues are critical for learning and avoiding predators, and a failure to respond can have dramatic repercussions for survival and recruitment.

## Introduction

Global Environmental Change (GEC) is having major impacts on all of the world's ecosystems and is viewed as one of the biggest threats to the natural world (Meehl et al. 2007). The earth's climate is warming at a far greater rate than at any time during the past 10,000 years, in part, due to greatly increased emissions of atmospheric CO_2_ (Walther et al. 2002). On a population level, GEC is expected to reduce both species abundance and diversity, in some cases resulting in local or even global extinctions (Hughes 2000; Williams et al. 2003; Munday 2004; Parmesan 2006). In addition to human-induced threats, animals are continually exposed to a broad array of risks and dangers in their natural environment. The number of dangers an animal will face throughout its life are numerous and varied (e.g., parasites, bacterial infections, con- and hetero-specifics), but one threat that may end in instant death if ignored is predation (Sih 1984; Kavaliers and Choleris 2001). It is the decisions that individuals make under the threat of predation that decide their fate and the genes they hold, in this way indirectly shaping prey community composition (Abrams 2000). Predators and their prey must continuously react and adapt to their environment, but in today's changing world, we know very little about how climate-induced habitat change will affect the intricate, and at times subtle, relationships between predators and their prey (Ferrari et al. 2011a).

Impacts of GEC on the marine ecosystem include rising sea surface temperatures, changing hydrodynamic regimes, and altered ocean chemistry (Munday et al. 2009; Roessig et al. 2004). In the ocean, coral reefs are among those habitats that are most likely to be adversely affected by climate change (Hughes et al. 2003; Hoegh-Guldberg et al. 2007). Coral reef environments represent one of the world's most biologically diverse ecosystems; however, very little is known of the interactions between predators and their prey that have shaped this astonishing biodiversity. Although these habitats have become popular systems for examining various aspects of the effect of climate change on behavioral interactions, the subject is still very much in its infancy (e.g., McCormick 2009; Dixson et al. 2010; Munday et al. 2010; Ferrari et al. 2011a,b). Decreases in ocean pH along with increases in water temperatures and the prevalence of severe storms have lead to bleaching and death of the live hard corals that underpin coral reef ecosystems (Hughes et al. 2003). As coral reefs degrade from live healthy coral to rubble, fish diversity and abundance declines (Graham et al. 2006). The majority of adult reef fishes are not directly dependent on live corals for survival (Pratchett et al. 2008). Despite this, whole fish communities have seen dramatic declines following loss of coral cover suggesting a widespread reliance on the coral reef habitat (Jones et al. 2004). The wider effects of coral bleaching on fish communities and, in particular, on the complex interrelationships between predators and prey remain poorly understood and research is required to identify the underlying behavioral processes that are driving the declines in abundance of fishes.

Coral reef fishes have complex life histories incorporating a widely dispersive larval phase, lasting from weeks to months, followed by settlement to the benthic reef environment. During this larval–juvenile transition, mortality rates are extremely high, primarily driven by predation (more than 50% are preyed upon in the first 48 h; Almany and Webster 2006). Successful identification of predators requires the newly settled larvae to detect olfactory and visual signs of danger all within a highly complex environment containing numerous different stimuli. Olfaction is particularly important at night when the larvae settle and in the highly complex habitats of coral reefs that limit visual abilities and assist cryptic predators (McCormick 2009; Vail and McCormick 2011). At this time, chemical alarm cues from the damaged skin of prey play an important role in the identification and avoidance of predators (Leduc et al. 2010; Lönnstedt et al. 2012). Recent studies have suggested that GEC is threatening to perturb the delicate balance between predators and their prey (Ferrari et al. 2011b). Munday et al. (2010) found that newly settled damselfish (*Pomacentrus wardi*) that had their olfactory sense disrupted through exposure to increased CO_2_ levels had a five to ninefold increase in mortality than control fish when placed on the reef. Similarly, Ferrari et al. (2011b) showed a five to sevenfold increase in mortality for another damselfish (*P. chrysurus*) exposed to elevated CO_2_. Furthermore, it has been suggested that coral dwelling damselfish (family Pomacentridae) are more susceptible to predation in bleached coral as the ability of prey fish to camouflage is diminished due to the increased perception of colorful prey fishes against the white background of the coral (Coker et al. 2009; McCormick 2009, 2012).

The goal of this study was to determine how predator risk assessment abilities of a naïve coral reef fish prey (*Pomacentrus amboinensis*) were affected by three different coral reef habitats, which represent a cline from healthy to degraded coral. Specifically, we undertook laboratory and field experiments to examine whether three different stages of coral (live healthy, thermally bleached, or degraded algae-covered dead coral) affected prey responses to: (1) conspecific damage-released chemical cues, (2) visual cues of a predator, and (3) a combination of visual and chemical cues. Further experiments addressed the mechanisms responsible for the impaired chemosensory responses in degraded coral habitats. Evidence suggests that the process of coral degradation will not only affect prey directly through changes in their resource base, but indirectly through modifications of the cues they use to assess predation risk.

## Methods

### Study species and collections

Experiments were conducted at Lizard Island (14°40′S, 145°28′E), northern Great Barrier Reef (GBR), Australia from October to November 2010. The ambon damselfish, *Pomacentrus amboinensis*, were used as a model prey species in all experimental trials. *P. amboinensis* is a common fish within coral reef fish communities in the Indo-Pacific (especially on the GBR) and settle to a wide range of habitats, but are found in highest densities in shallow sandy areas on live corals (McCormick et al. 2010). Their pelagic larval phase lasts between 15 and 23 days and the new recruits are readily collected overnight with light traps that have been moored just outside the reef (see Meekan et al. 2001 for design). Fish used for the present studies were all caught in light traps and brought back to the Lizard island research station at dawn and placed in 60-L flow-through seawater holding tanks (densities of ∼50 fish/tank). Fish were fed twice daily with newly hatched *Artemia* sp. nauplii ad libitum to allow for recovery from the stress of capture. Juvenile *Apogon doederleini* were used as control fish for adding the skin extract cue of a heterospecific fish into the aquarium. These fish are phylogenetically and ecologically distant from *P. amboinensis,* thus being an ideal control fish. Apogonids were collected on the reef using hand nets.

One of the most common and abundant predators on new settlers during the recruitment season is the dottyback *Pseudochromis fuscus* (Feeney et al. 2012). As naïve prey fish have been found to have an innate fright reaction to the sight of this predator (unpublished data), it was used as a model predator species to expose fish to in the various habitats. As a control fish for the visual cues, we used the herbivorous goby (*Amblygobius phalanea)*, which are of similar size and shape as adult dottybacks. Both of these species are found in large numbers around Lizard Island and were collected using hand nets and a dilute solution of clove oil anesthetic. Gobies and dottybacks were brought back to the research station and placed individually in 13-L aquaria and fed daily with fish food pellets.

Live healthy and dead algae-covered hard coral (*Pocillopora damicornis*) were collected from the fringing reefs around Lizard Island and placed in well aerated 500-L flow-through seawater holding tanks. The process of bleaching involves the expulsion of symbiotic zooxanthellae algae when the coral is under stress. This can happen when water temperatures reached >1°C above the summer maximum (Anthony et al. 2007). *Pocillopora damicornis* colonies bleach in about 10 days and will die in 2–3 weeks if the temperature remains consistently high, after which they get rapidly colonized by various algal and invertebrate species. In this study, healthy colonies were thermally bleached over a 12-day period using the protocol of McCormick et al. (2010). After colonies had expelled their zooxanthellae and were visibly bleached, but not dead, temperatures were once again lowered to the ambient 28°C.

### Experimental outline

We conducted three separate experiments, two in the laboratory and one in the field. All experiments were designed to test the effects of coral degradation on antipredator response of fishes to predation cues. The first experiment, conducted in the laboratory, examined responses of damselfish to visual, chemical, and combined visual and chemical cues that indicate risk. The second experiment, conducted in the field, focused solely on responses to chemical information and was undertaken to determine the extent to which the findings of the first study were pertinent to natural populations. The final laboratory experiment tested if seawater that contained, or had been in contact with, dead algae-covered coral caused a modification (alteration or degradation) of conspecific chemical alarm cues or simply masked (i.e., overwhelmed) alarm cues from being detected.

### Design of laboratory experiments

All behavioral observations were conducted in transparent 15-L aquaria (38 × 24 × 27 cm) with a constant flow of seawater until the commencement of trials (see Appendix S1 in supporting information). The tanks were set up, so they were continuously fed seawater from three separate reservoirs (60 L) that either contained four coral heads (10 × 15 × 12 cm) of live healthy, live thermally bleached, or dead algae-covered coral habitat of the common bushy hard coral *Pocillopora damicornis*. One of the three types of coral habitat (live healthy, live bleached, or dead coral) was placed along the short side of the aquaria creating vertical shelters (18 × 20 × 4 cm). All corals were replaced every 2 days and used coral was returned to the field. Naïve *P. amboinensis* (*n* = 15–17/treatment) were placed individually in the aquaria and allowed to acclimate overnight. Prior to the start of the trial, the water flow was stopped and 5 mL of *Artemia* sp (∼ 550 *Artemia*) was added to the aquaria to stimulate feeding. The behavior of a single *P. amboinensis* was recorded for a 4-min pre-stimulus period. Immediately following the pre-stimulus period, a further 5 mL of *Artemia* was added and fish were exposed to the relevant cue treatment and the behavior of the fish was then recorded for a further 4 min.

To prepare the damage-released cues, we sacrificed one recruit per trial using cold shock. The flank of each recruit was then superficially cut six times. The total cue area was rinsed with 10 mL of seawater that had been collected from the test aquaria and was then filtered through filter paper (47 mm Ø) prior to being used in the experiment. The behavioral response to experimental treatments was quantitated by recording: total number of successful feeding strikes, total time spent inside of shelter (s), and activity (quantitated as the number of times a fish crossed a line on the grid (3 × 3 cm) that had been drawn on the side of the tank).

### Experiment 1: Does coral degradation influence prey risk assessment in the laboratory?

Naïve fish placed individually within aquaria containing one of three coral habitats (live healthy, bleached, or dead algae-covered coral) were exposed to one of seven different cue treatments and their behavior was recorded as above (*n* = 15–16). Chemical cue treatments included: (1) damage-released chemical cue of injured conspecifics; (2) control cues from injured heterospecifics, *A. doederleini;* and (3) saltwater control. Visual treatments included: (4) a transparent bag filled with water; (5) a transparent bag that contained a herbivorous goby, *A. phalanea*; (6) a transparent bag that contained a predatory dottyback, *P. fuscus***.** The seventh treatment included a combination of a pairing of treatment one and six, as we reasoned that fish would have a stronger response when both sources of risk cues were available (e.g., Lima and Steury 2005; McCormick and Manassa 2008).

### Experiment 2: Does coral degradation influence the antipredator response to chemical indicators of risk in the field?

Our laboratory studies indicated that coral degradation influences the responses of damselfish to chemical cues that indicate risk. This experiment aimed to determine whether there was evidence of environmental masking or alteration of damage-released cues in the field under natural conditions. All experimental trials were conducted within a sand patch surrounded by hard coral reef (composed of a typical diversity of live and dead coral habitats) using SCUBA at depths between 4 and 8 m. Small patch reefs (25 × 15 × 20 cm) of either live healthy *P. damicornis*, thermally bleached *P. damicornis*, or dead algal-covered *P. damicornis* were assembled in the sandy area adjacent to the reef (see Appendix S2 in supporting information). To avoid any contamination between patch reefs, there was a minimum of 3 m between patches and we moved in an up-current direction when doing the experiment. A single juvenile *P. amboinensis* was placed onto each patch reef and allowed to acclimate for a minimum of 30 min before behavioral observations commenced. A 2-m plastic tube was attached up-current at the edge of the patch reef using metal skewers. The behavioral response of naïve *P. amboinensis* to three different treatments was tested: (1) skin extracts from damaged conspecifics; (2) skin extracts from damaged heterospecifics; and (3) saltwater (blank control) (*n* = 15). The behavior of focal fish was quantified for 3 min before (pre-stimulus period) and 3 min after (post-stimulus period) the addition of a stimulus (skin extract or saltwater).

To prepare skin extracts underwater, light trap caught *P. amboinensis* fish were brought underwater in 75 × 125-mm click seal bags, which were filled with ∼ 40 mL of sea water. Fish were euthanized by a quick blow to the brain case and the epidermis of the fish was lightly scratched using a scalpel blade that had been placed in the bag. A disposable syringe equipped with a fine needle was used to perforate the bag and extract 30 mL of the prepared stimulus. Behavior of the fish was assessed by a SCUBA diver positioned at least 1.5 m away from the patch reef. Four aspects of activity and behavior were estimated for each 3-min sampling period: bite rate (successful and unsuccessful strikes), average distance from shelter (cm), maximum distance from shelter (cm), and time spent in shelter (s). Three minutes has previously been found to be sufficient to obtain a representative estimate of an individual's behavior (bite rate), which also relates strongly to survival in the wild at this life stage (McCormick and Meekan 2010). Distance from shelter for these recently settled fishes has also been found to be closely related to survival in the first few days after settlement to the reef (McCormick 2009, 2012; McCormick and Meekan 2010; Munday et al. 2010).

### Experiment 3: Does dead coral mask or modify chemical indicators of predation risk?

Here, we attempted to identify a possible mechanism responsible for the impaired responses that we observed for fish exposed to alarm cues in dead coral habitats. Specifically, we tested whether the impaired chemosensory responses in dead coral likely resulted from (1) a chemical alteration/degradation of the cue (i.e., a structural change in the chemical cues that are not reversible), or (2) odor masking, whereby the lack of a behavioral response in dead coral occurs as a result of a high level of background odor that overwhelms the fish's olfactory sense making the cues hard to discern. To accomplish this, individual naïve fish (*n* = 16–19) were placed in tanks containing one of two habitats (live or dead hard coral) and left to acclimate. Fish in each habitat were then exposed to conspecific skin extracts that had been prepared (as above) with water from two different sources: (1) water that had flown past dead corals (from a 60-L flow-through tank containing four dead, algae-covered colonies of *P. damicornis* [10 × 15 × 12 cm]); or (2) water that had flown past live healthy *P. damicornis* (four colonies in a 60-L tank). Their behavior was recorded before and after the injection of the stimulus as above (c). In accordance with the previous experiments, we predicted impairment in behavioral responses for fish exposed to alarm cues prepared in healthy coral water, but tested in dead coral habitats, and for fish exposed to alarm cues prepared in dead coral water and tested in dead coral habitats. We predicted fish exposed to alarm cues prepared from healthy coral water and then tested in healthy coral habitat would display antipredator responses. If alarm cues are altered/degraded by chemicals released from the dead coral and these changes are not reversible, then fish tested in healthy live coral environments should fail to respond to alarm cues prepared in dead coral water. In contrast, if fish exposed to alarm cues prepared in dead coral water and tested in the presence of live coral respond normally, then this would be regarded as evidence that the dead coral water simply masks the odor of the alarm cues, as the effect is reversible with dilution into the tank.

### Statistics

To test whether the behavior of fish differed (in both the field and the laboratory) among the three different habitats (healthy, bleached, and dead coral), and whether fish had been given olfactory indicators of risk (conspecific skin extract, heterospecific skin extract, or a saltwater control), visual indicators of risk (visual predator, visual herbivore, or none), or a combination of visual (predator) and chemical indicators of risk (conspecific chemical alarm cue), a multivariate analysis of variance (MANOVA) was employed. A two-way MANOVA tested whether the behavior of fish differed between the background habitat (live or dead coral) or how the skin extract cue had been prepared (mixed with water in that had been in contact with live healthy coral or dead coral) and whether behavior was affected by the interaction between these two factors. All data were analyzed as the difference between the magnitude of behaviors before an experimental stimulus and after exposure to a stimulus (post-pre). Variables included in the analysis were as follows: bite rate, activity level, distance from shelter, and time spent in shelter. Time spent in shelter was log_10_(x + 1) transformed to meet assumptions of normality. Univariate analysis of variance (ANOVA) was employed to examine the nature of the significant difference found by MANOVAs. Significant ANOVAs were further explored using unequal sample Tukey's HSD tests. A reduction in activity and foraging and movement into or close to the shelter are common antipredator responses of damselfish to risk in both the laboratory and field (Lönnstedt et al. 2012).

## Results

**Figure 1:**
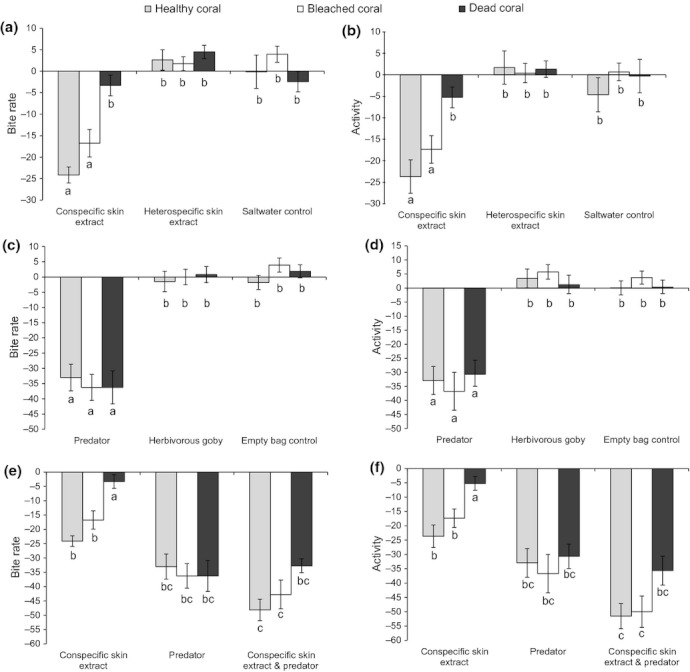
Coral degradation affects assessment of predation risk by prey in the laboratory; with the column on the left showing the mean change in bite rate, and the column on the right displays the mean change in activity of fish to the different treatments. Change in behavioral responses of coral reef fish exposed to chemical alarm cues (*a,b*), a predator (*c,d*), and a pairing of the two (*e, f*). Bite rates and activity levels are significantly decreased when exposed to threat cues in both healthy and bleached coral habitats. When exposed to the visual sight of a predator activity, levels and bite rates were strongly reduced regardless of the background habitat. In the healthy and bleached habitats, these behaviors were intensified when fish were presented with the sight of a predator paired with a chemical cue. Letters above or below bars represent Tukey's HSD grouping of means (*α* = 0.05).

The way fish changed their behavior in response to conspecific damage-released cues differed among habitats in the laboratory compared with the two controls (MANOVA: Pillai's Trace = 0.19, degrees of freedom [df] = 8, 266, *P* < 0.001). Fish exhibited a significant decrease in bite rate when exposed to chemical cues in both the healthy and bleached coral habitats (Tukey's HSD tests: *P* < 0.05; Fig. 1a). Fish in the dead coral habitat did not significantly change their bite rate compared with the controls when exposed to damage-released chemical cues (Tukey's HSD tests: *P* > 0.05; Fig. 1a). Fish decreased foraging and activity in the three different habitats when exposed to the sight of a predator compared with the two visual controls (MANOVA: Pillai's Trace = 0.17, df = 8, 266, *P* < 0.0001; Fig. 1c,d).

Habitat type strongly influenced the response of fish to chemical (conspecific skin extract), visual (visual predator), or a combination of chemical and visual predator cues in the laboratory (Pillai's Trace = 0.19, df = 8, 266, *P* < 0.005). Univariate ANOVAs that examined the change in behavior after exposure to the various threat cues showed that there was a significant difference in bite rate, activity, and time spent in shelter depending on which habitat the fish occupied (*P* < 0.01; Fig. 1e,f). Fish in healthy and bleached habitats strongly reduced both activity levels and bite rates to visual and chemical threat cues, and there was an additive effect when both cue sources were present. Prey fish did not respond stronger to the simultaneous exposure of both sources of risk in the dead coral compared with both the live and bleached habitats (*P* > 0.05; Fig. 1e,f).

**Figure 2:**
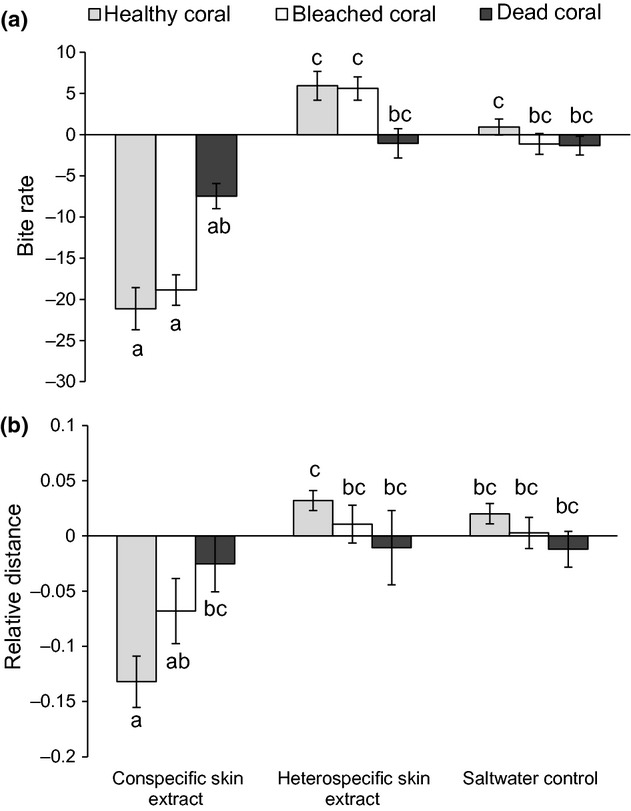
Mean change of naïve fish when exposed to various olfactory cues in the field. (a) Bite rate is strongly reduced in both healthy and bleached coral when exposed to conspecific skin extracts while not when exposed to heterospecific skin extracts or a saltwater control. (b) When exposed to chemical alarm cues of conspecifics, fish strongly reduced their distance from shelter in the live healthy coral, but tended to retire to shelter less in both bleached and dead coral habitats. Letters above or below bars represent Tukey's HSD grouping of means (*α* = 0.05).

The habitat fish were on affected their response to damage-released chemical alarm cues in the field (MANOVA: Pillai's Trace = 0.35, df = 4,123, *P* < 0.001; Fig. 2). Univariate statistics indicate that prey fish were negatively impacted in dead coral habitats when assessing predation risk by olfaction. In the healthy habitats, fish responded to chemical cues by retreating to shelter and reducing their foraging compared with the controls (Tukey's HSD tests: *P* < 0.05; Fig. 2). Although fish responded to damage-released cues when in the bleached coral and fish spent less time inside the habitat, their behavior did not significantly differ from the two controls (Fig. 2b; *P* < 0.05). In the dead coral habitat, fish did not significantly change their behavior when exposed to damage-released cues compared with the controls (Tukey's HSD tests: *P* > 0.05; Fig. 2).

**Figure 3:**
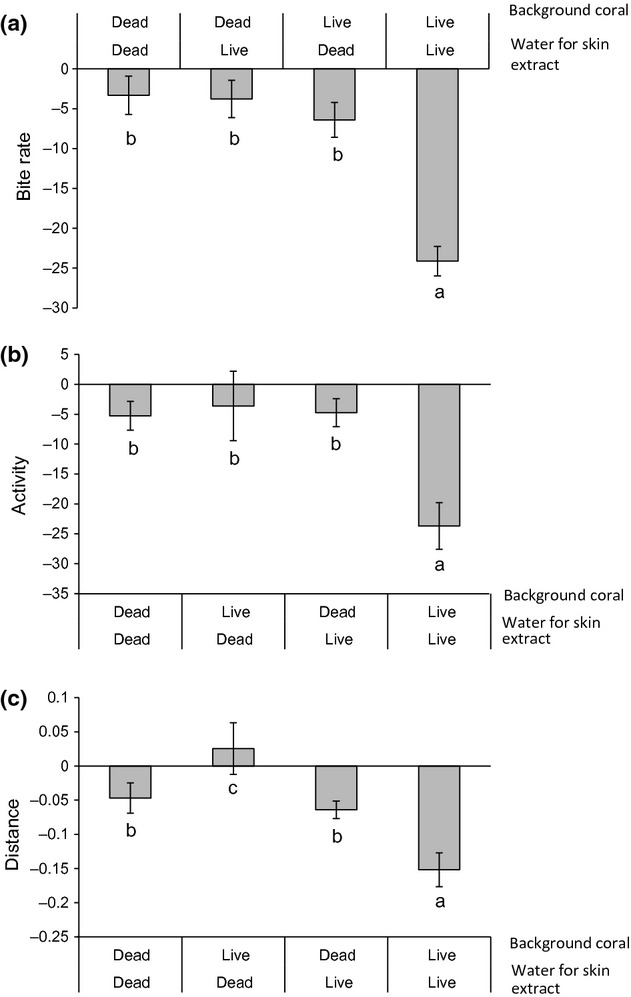
Comparison of the behavior of *Pomacentrus amboinensis* in the laboratory that had been exposed to conspecific skin extracts prepared with water containing either live healthy coral or dead algae-covered coral in one of two background habitats (live healthy or dead algae-covered). Behaviors are the change between the 4-min pre- and post-stimulus period in (*a*) bite rate, (*b*) activity level, and (*c*) average distance from the coral shelter. Letters above or below bars represent unequal Tukey's HSD grouping of means (*α* = 0.05).

There was a strong interactive effect of background habitat and the type of water that the cue was prepared with on the behavior of naïve fish (Pillai's Trace = 0.4, df = 3,59, *P* < 0.001; fig. 3a,b). This was caused by the combination of a live healthy coral background and skin extract cues prepared with water that had been in contact with live coral differing from all the other treatments, which in turn, did not differ from one another (fig. 3a,b). Univariate ANOVAs on each behavioral variable revealed that naïve fish in tanks with a background of healthy live coral responded with a reduction in activity, bite rate, and distance from shelter (F_1,61_ = 17.7, *P* < 0.001; F_1,61_ = 11.9, *P* ≤ 0.001; F_1,61_ = 18.7, *P* < 0.001) when exposed to conspecific skin extracts that had been prepared with seawater that had only been in contact with live healthy coral (fig. 3a,b). Contrastingly, fish with a background habitat consisting of dead, algae-covered coral did not respond to any skin extracts (regardless of how they had been prepared). Similarly, fish in live healthy coral habitats did not respond to conspecific skin extracts prepared with water that had been in contact with degraded coral habitats. It appears as though seawater that is, or has been in contact with, dead algae-covered coral may alter the structure of conspecific chemical alarm cues.

## Discussion

We showed that coral degradation had a profound influence on the behavioral responses of fish to cues that indicate predation risk. Fish in live healthy and bleached coral fed above the colony and reduced swimming, ignored food, and sought refuge when exposed to either chemical or visual indicators of risk. Prey in the dead, algae-covered coral habitat showed a similar antipredator response when exposed to the sight of a predator, but when presented with damage-released alarm cues of conspecifics, they did not visibly change their behavior in either the laboratory or the field. While a pairing of olfactory and visual threat cues had an additive effect on the prey response in live coral habitats, prey occupying degraded habitats did not show a stronger response when given the combined cue sources. Failing to respond to an olfactory indicator of risk greatly increases the likelihood of being preyed upon (Munday et al. 2010; Ferrari et al. 2011b). Fish with impaired olfactory abilities are also less likely to find a suitable settlement sites and potential mates (Curtis et al. 2001; Dixson et al. 2010; Munday et al. 2009, 2010; Devine et al. 2012).

We know from previous studies that small bodied coral dwelling damselfish (family Pomacentridae) decline in abundance following coral bleaching and reef degradation (Wilson et al. 2006; McCormick 2009). As they are not obligate corallivores, it has been not clear why they exhibit such strong reductions in abundance following large scale bleaching events. It was initially believed to be due to a decline in coral cover and the subsequent reduction in the structural complexity of the coral reef framework (making them more susceptible to predators), but bleaching does not necessarily equate to a loss in habitat structure in the short term (Pratchett et al. 2008). During bleaching, the density of zooxanthellae (photosynthetic algae within the coral tissue) is reduced either through the expulsion or death of the minute algal cells, thus not affecting the structure, but only the pigmentation of the coral. It is the subsequent death and erosion that results in the loss of coral structure (Booth and Beretta 2002). Hence, it is the live coral in itself that offers some sort of advantage to fish. This study demonstrates that fish appear unwilling to retreat back into bleached or degraded coral when exposed to threat cues, spending less time in shelter compared with when occupying the live healthy coral colonies. McCormick (2009) suggested that the smell of dying tissue may force recruit stage fish away from bleached coral, leading to higher vulnerability. Our results suggest that the mechanism underlying the move away from degraded coral habitats may be their reduced ability to identify the olfactory cues that are innately associated with predation threat (the chemical alarm cues). The information on which they base their decision has changed, affecting their perception of where they should best sit along the axis of risk from shelter (and reduced foraging opportunities) to open water (and increased foraging opportunities).

The relative context in which a threat stimulus is received can influence both the quality and effectiveness of a signal as certain environmental conditions, or “background noise”, can alter the signals perceived form (Endler 1992). The phenomenon of odor masking has been well studied in terrestrial environments (for a comprehensive review see Schroder and Hilker 2008), but the focus in this literature is often background odors masking resource indicating cues. For instance, certain plants produce an odor that repels insects, or hides the odors of their host plants (Mauchline et al. 2005). They benefit the plants by allowing them to effectively hide from consumers in a complex chemical environment. In our study, we tested whether the background odor of dead coral masked or modified the scent of alarm cues, reducing the response of prey to threats. Once a coral is dead and overgrown by algae, a whole new community settles into it and all these different life forms (together with the algae) may overpower other odors in the environment (such as the scent of wounded conspecifics). However, we found no evidence for odor masking, as fish exposed to alarm cues prepared in dead coral water did not elicit a response in water containing healthy coral. We prepared the alarm cue in 10 mL of water and injected the cue into a tank containing 15 L of water. Despite this huge dilution effect, the “unmasked cue” did not elicit a fright response in the fish. Fish have been shown to have a remarkable ability to differentiate between threat cues even when presented together (Mitchell et al. 2011), which also suggests that odor masking is unlikely. As an alternative to odor masking, our results support the hypothesis that dead coral rapidly alters or degrades the chemical alarm cue. Whenever the alarm cues were in contact with dead coral (either prepared in dead coral water or injected into a tank containing dead algae-covered coral), fish failed to elicit normal antipredator responses. As such, our results resemble the responses of salmonid fishes in freshwater systems, whereby the alarm cues are rendered inactive when the pH drops to 6.0 (Leduc et al. 2004). The proximate chemical mechanism responsible for this change in our system remains unknown, but likely is not a result of a pH change, as this was not altered in the study systems given that marine systems do not show large changes in pH (Gagliano et al. 2010).

The impact on the olfactory sense due to degraded habitat is different from the recently documented impacts of elevated dissolved CO_2_ on the olfactory sense. Dissolved CO_2_ elevated above 900 μatm has been shown to alter the function of neurotransmitters in fish (Nilsson et al. 2012), leading to the reduced discrimination of pertinent sensory cues (Dixson et al. 2010; Ferrari et al. 2012b) and the negation of learning processes associated with the correct identification of chemical alarm cues (Ferrari et al. 2012a,b). Luckily, there may be sufficient variability in the physiological response at low CO_2_ concentrations (700 *μ*atm) within populations for fish to adapt to this CO_2_-rich world through ecological selection. In contrast, the mechanism described in this study is external to the animal, and involves the modification of the cue, such that it is either not recognized or inappropriately categorized. Our data suggest that all individuals where similarly impacted, suggesting a limited ability to adapt to the loss of this important sensory cue. As coral death and degradation becomes increasingly prevalent (Wilkinson 2004), further research is required to determine the extent to which the risk assessment of other species may be affected by the same mechanism and the community wide repercussions.

Due to GEC, coral reefs all over the world are declining in health and what once were fields of live coral are now low lying rubble beds. Fish living within these changed environments are more likely to become stressed as the coral degrades, both as a result of the loss of refuge space and from a change in their olfactory environment. Is it plausible that the fish are so stressed by their new surroundings that they fail to respond to predator threats? This seems very unlikely as the fish still responded to the sight of a predator when in the dead coral. Predation is one of the most important processes shaping coral reef fish communities. Our current findings suggest that coral bleaching and coral death will impact the crucial interactions between fish predators and their prey. Bleached and dead coral patches appear to interfere with olfactory cues critical for the assessment of risk by prey. Without detecting the olfactory signposts of risk, prey are unable to identify the early signs of danger and are more likely to fall prey to hungry predators (McCormick 2009; Munday et al. 2010). Biologists and managers wishing to predict the long-term consequences of global environmental change on reef fish assemblages will need to understand the repercussions of this crucial developmental bottleneck (Lönnstedt et al. 2012; Ferrari et al. 2012a,b).

## Supporting Information

**Appendix S1.** Outline of the experimental set-up in the laboratory

**Appendix S2.** Outline of the experimental set-up in the field

## References

[b1] Abrams PA (2000). The evolution of predator-prey interactions: theory and evidence. Annu. Rev. Ecol. Syst.

[b2] Almany GR, Webster MS (2006). The predation gauntlet: early post-settlement mortality in reef fishes. Coral Reefs.

[b3] Anthony KRN, Connolly SR, Hoegh-Guldberg O (2007). Bleaching, energetics, and coral mortality risk: effects of temperature, light, and sediment regime. Limnol. Oceanogr.

[b4] Booth DJ, Beretta GA (2002). Changes in a fish assemblage after a coral bleaching event. Mar. Ecol. Prog. Ser.

[b100] Coker DJ, Pratchett MS, Munday PL (2009). Coral bleaching and habitat degradation increase susceptibility to predation for coral-dwelling fishes. Behav. Ecol.

[b5] Curtis JT, Liu Y, Wang Z (2001). Lesions of the vomeronasal organ dirupt mating-induced pair bonding in female prairie voles (*Microtus ochrogaster**Brain*. Res.

[b6] Devine BM, Munday PL, Jones GP (2012). Homing ability of adult cardinalfish is affected by elevated carbon dioxide. Oecologia.

[b7] Dixson DL, Munday PL, Jones GP (2010). Ocean acidification disrupts the innate ability of fish to detect predator olfactory cues. Ecol. Lett.

[b8] Endler JA (1992). Signals, signal conditions, and the direction of evolution. Am. Nat.

[b9] Feeney WE, Lonnstedt OM, Bosiger Y, Martin J, Jones GP, Rowe RJ (2012). High rate of prey consumption in a small predatory fish on coral reefs. Coral Reefs.

[b10] Ferrari MCO, Dixson DL, Munday PL, McCormick MI, Meekan MG, Sih A (2011a). Intrageneric variation in anti-predator responses of coral reef fishes to ocean acidification: implications of projecting climate change on marine communities. Glob. Change Biol.

[b11] Ferrari MCO, McCormick MI, Munday PL, Meekan MG, Dixson DL, Lonnstedt OM (2011b). Putting prey and predator into the CO2 equation - quantitative and qualitative effects of ocean acidification on predator-prey interactions. Ecol. Lett.

[b12] Ferrari MCO, Manassa R, Dixson DL, Munday PL, McCormick MI, Meekan MG (2012a). Effects of ocean acidification on learning in coral reef fishes. PLoS ONE.

[b13] Ferrari MCO, Munday PL, McCormick MI, Meekan MG, Dixson DL, Lonnstedt OM (2012b). Effects of ocean acidification on visual risk assessment in coral reef fishes. Funct. Ecol.

[b14] Gagliano M, McCormick MI, Moore JAY, Depczynski M (2010). The basics of acidification: baseline variability of pH on Australian coral reefs. Mar. Biol.

[b15] Graham NAJ, Wilson SK, Jennings S, Polunin NVS, Bijoux JP, Robinson J (2006). Dynamic fragility of oceanic coral reef ecosystems. Proc. Nat. Acad. Sci.

[b16] Hoegh-Guldberg A, Mumby PJ, Hooten AJ, Steneck RS, Greenfield P, Gomez E (2007). Coral reefs under rapid climate change and ocean acidification. Science.

[b17] Hughes L (2000). Biological consequences of global warming: is the signal already apparent? *Trends Ecol*. Evol.

[b18] Hughes TP, Baird AH, Bellwood DR, Card M, Connolly SR, Grosberg CFR (2003). Climate change, human impacts, and the resilience of coral reefs. Science.

[b19] Jones GP, McCormick MI, Srinivasan M, Eagle JV (2004). Coral decline threatens fish biodiversity in marine reserves. Proc. Nat. Acad. Sci. U.S.A.

[b20] Kavaliers M, Choleris E (2001). Antipredator responses and defensive behaviour: ecological and ethological approaches for the neurosciences. Neurosci. Behav. Rev.

[b21] Leduc AOHC, Kelly JM, Brown GE (2004). Detection of conspecific alarm cues by juvenile salmonids under neutral and weakly acidic conditions: laboratory and field tests. Behav. Ecol.

[b22] Leduc AOHC, Kim JW, MacNaughton CJ, Brown GE (2010). Sensory complement model helps to predict diel alarm response patterns in juvenile Atlantic salmon (*Salmo salar*) under natural conditions. Can. J. Zool.

[b23] Lima SL, Steury TD (2005). The perception of predator risk - the foundation of non-lethal predator-prey interactions. In Ecology of Predator/Prey Interactions (eds. Barbosa P., Castellanos I.).

[b24] Lönnstedt OM, McCormick MI, Meekan MG, Ferrari MCO, Chivers DP (2012). Learn and live: the role of predator experience in influencing prey behaviour and survival. Proc. R. Soc. Lond. B Biol. Sci.

[b25] Mauchline AL, Osborne JL, Martin AP, Poppy GM, Powell W (2005). The effects of non-host plant essential oil volatiles on the behaviour of the pollen beetle *Meligethes aeneus*. Entomol.

[b26] McCormick MI (2009). Behaviourally mediated phenotypic selection in a disturbed coral reef environment. PLoS ONE.

[b27] McCormick MI (2012). Lethal effects of habitat degradation on fishes through changing competitive advantage. Proc. R. Soc. Lond. B. Sci.

[b28] McCormick MI, Manassa R (2008). Predation risk assessment by olfactory and visual cues in a coral reef fish. Coral Reefs.

[b29] McCormick MI, Meekan MG (2010). The importance of attitude: the influence of behaviour on survival at an ontogenetic boundary. Mar. Ecol. Prog. Ser.

[b30] McCormick MI, Moore JAY, Munday PL (2010). Influence of habitat degradation on fish replenishment. Coral Reefs.

[b31] Meehl GA, Stocker TF, Collins P, Friedlingstein P, Gaye AT, Gregory JM, Solomon S, Qin D, Manning M, Chen Z, Marquis M, Averyt KB (2007). Global climate projection. Climate Change 2007: The Physical Science Basis. Contribution of Working Group i to the Fourth Assessment Report on the Intergovernmental Panel on Climate Change.

[b32] Meekan MG, Wilson SG, Halford A, Retzel A (2001). A comparison of catches of fishes and invertebrates by two light trap designs, in tropical NW Australia. Mar. Biol.

[b33] Mitchell M, McCormick MI, Ferrari MCO, Chivers DP (2011). Coral reef fish rapidly learn to identify multiple unknown predators upon recruitment to the reef. PLoS ONE.

[b34] Munday PL (2004). Habitat loss, resource specialization, and extinction on coral reefs. Glob. Change Biol.

[b35] Munday PL, Dixson DL, Donelson JM, Jones GP, Pratchett MS, Devitsina GV (2009). Ocean acidification impairs olfactory discrimination and homing ability of a marine fish. Proc. Nat. Acad. Sci. U.S.A.

[b36] Munday PL, Dixson DL, McCormick MI, Meekan MG, Ferrari MCO, Chivers DP (2010). Replenishment of fish populations is threatened by ocean acidification. PNAS.

[b37] Munday PL, McCormick MI, Nilsson GE Impact of global warming and rising CO_2_ on coral reef fishes: what hope for the future?. J. Exp. Biol.

[b38] Munday PL, McCormick MI, Meekan MG, Dixson DL, Watson S, Ferrari MCO Selection for CO_2_ tolerance in marine fishes. Ocean Acidification.

[b39] Nilsson GE, Dixson DL, Domenici P, McCormick MI, Sørensen C, Watson S (2012). Near-future CO2 levels alter fish behaviour by interference with neurotransmitter function. Nature Climate Change.

[b40] Parmesan C (2006). Ecological and evolutionary responses to recent climate change. Annu. Rev. Ecol. Evol. Syst.

[b41] Pratchett MS, Munday PL, Wilson SK, Graham NAJ, Cinner JE, Bellwood DR (2008). Effects of climate-induced coral bleaching on coral-reef fishes ecological and economical consequences. Oceanogr. Mar. Biol. Annu. Rev.

[b42] Roessig JM, Woodley CM, Joseph JC, Hansen LJ (2004). Effects of global climate change on marine and estuarine fishes and fisheries. Rev. Fish Biol. Fish.

[b43] Schroder R, Hilker M (2008). The relevance of background odor in resourcse location by insects: a behavioural approach. BioSci.

[b44] Sih A (1984). The behavioural response race between predator and prey. Am. Nat.

[b45] Vail A, McCormick MI (2011). Larval reef fish avoid predator scent when choosing a home. Biol. Let.

[b46] Walther GR, Post E, Convey P, Menzel A, Parmesan C, Beebee TJC (2002). Ecological responses to recent climate change. Nature.

[b47] Wilkinson CR (2004). Status of coral reefs of the world 2004, vols 1 and 2.

[b48] Williams SE, Bolitho EE, Fox S (2003). Climate change in Australian tropical rainforests: an impending environmental catastrophe. Proc. R. Soc. Lond. B Biol. Sci.

[b49] Wilson SK, Graham NAJ, Pratchett MS, Jones GP, Polunin NVC (2006). Multiple disturbances and the global degradation of coral reefs: are reef fishes at risk or resilient?. Glob. Change Biol.

