# Erratum

**DOI:** 10.1002/ece3.4172

**Published:** 2018-05-24

Erratum for: Lonnstedt OM, McCormick MI, Chivers DP (2013) Degraded environments alter prey risk assessment. Ecology and Evolution 3:38–47: https://onlinelibrary.wiley.com/doi/full/10.1002/ece3.388

Subsequent to the publication of this article, the Editorial team became aware that the data for this paper had not been made available on a public repository, contrary to the requirements of our journal policies (https://onlinelibrary.wiley.com/page/journal/20457758/homepage/forauthors.html).

The authors have now made their data publicly available on Tropical Data Hub (https://tropicaldatahub.org/) accessible via https://doi.org/10.4225/28/5a7b86489e46b

