# Supplementary material for: Degraded Environments Alter Prey Risk Assessment
Source: Ecol Evol. 2013 Jan 10;3(1):38–47. doi: 10.1002/ece3.388 (PMC3568841; doi:10.1002/ece3.388)
Supplement: Supplementary file 1 [file ece30003-0038-SD1.pdf]

Online Supporting Information

<u>Appendix S1. Outline of the experimental set-up in the laboratory</u>

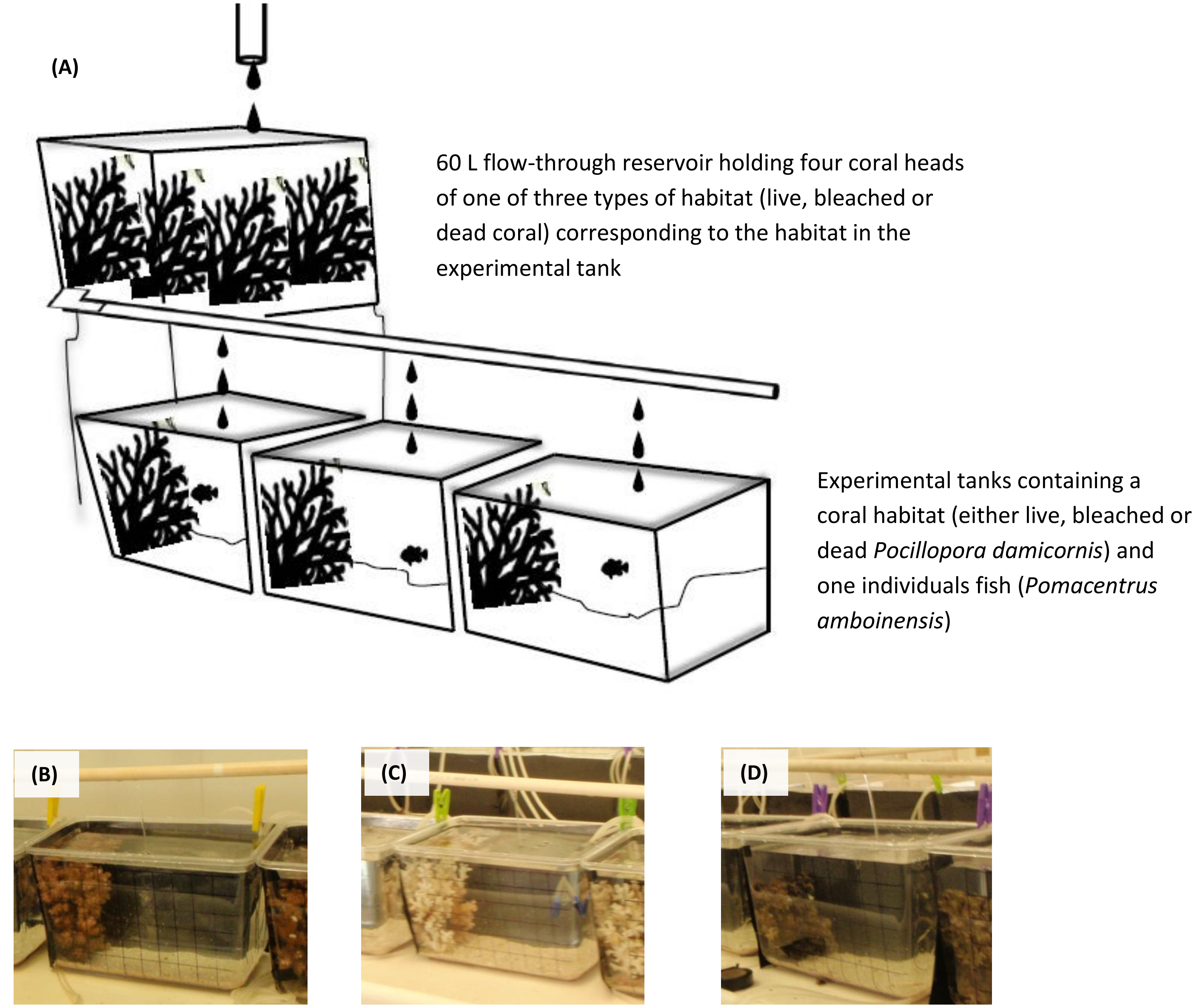

Figure S1. Outline of the experimental set-up in the laboratory (A). A 60 L reservoir held 4 coral heads (10x15x12cm) of live healthy (B), live thermally-bleached (C) or dead-algal-covered (D) coral habitat of the common bushy hard coral *Pocillopora damicornis*. There were three different reservoirs each corresponding to one of the three coral habitats. Water was flowing through the reservoir and into the experimental aquaria (15 L; 38x24x27cm) that held the corresponding coral habitat (18x20x4cm) to the reservoir and one individual fish (*Pomacentrus amboinensis*).

Appendix S2. Outline of the experimental set-up in the field

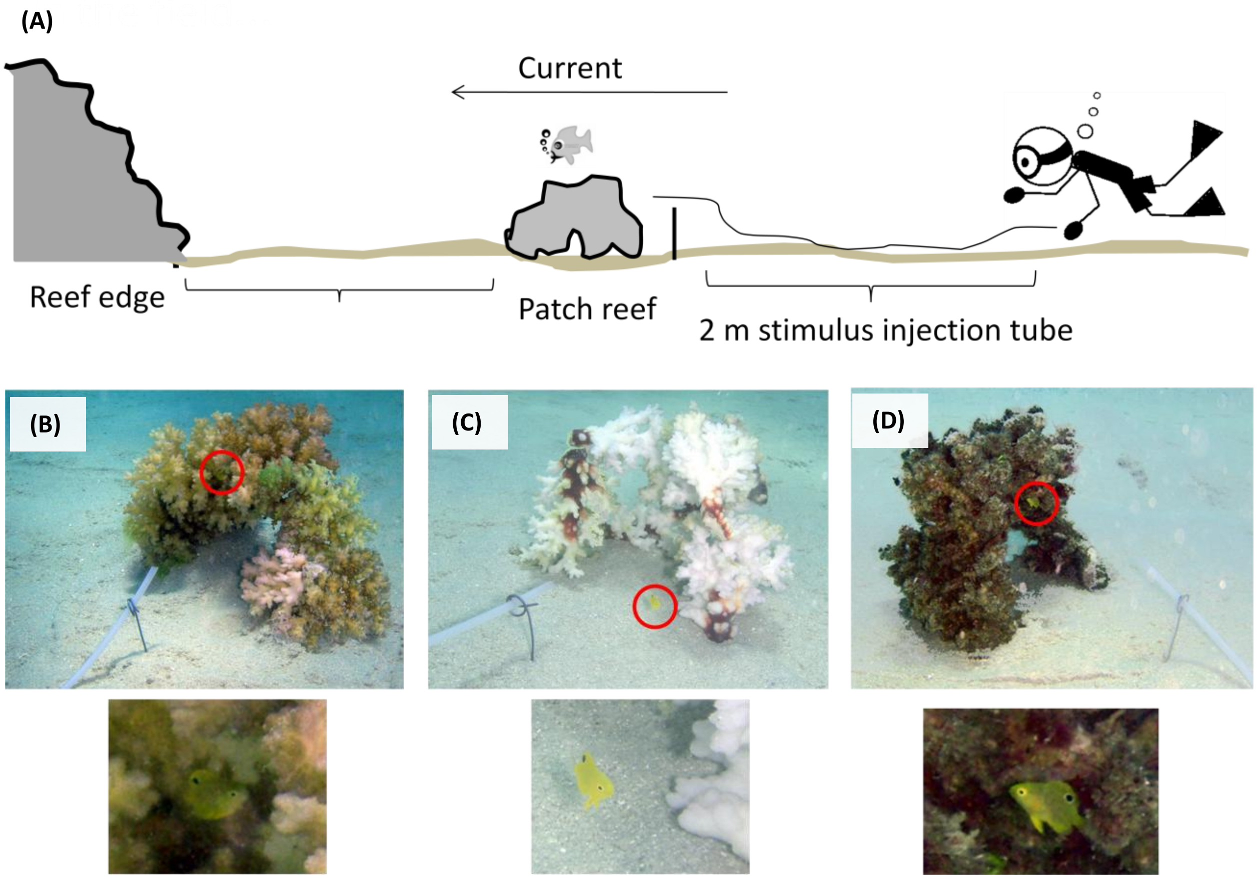

Figure S2. Experimental set-up in the field (A). Individual fish were placed on one of three coral patches ((B) live healthy, (C) live thermally-bleached or (D) dead-algal-covered coral). Fish were left to acclimate on the patch reef (25x15x20cm) and behavioural observations were commenced. A 2 m plastic tube was attached up-current at the edge of the patch reef using metal skewers. The behavioural response of naïve *P. amboinensis* to 3 different treatments was tested: skin extracts from damaged conspecifics; skin extracts from damaged heterospecifics and saltwater (blank control). The behaviour of focal fish was quantified for 3 min before (pre-stimulus period) and 3 min after (post-stimulus period) the addition of a stimulus (skin extract or saltwater). The red circle denotes the location of the fish on the patch reef. In order to correctly assess the behaviour of fish a magnifying glass was utilized.
