# Supplementary figures and images for: Degraded Environments Alter Prey Risk Assessment
Source: Ecol Evol. 2013 Jan 10;3(1):38–47. doi: 10.1002/ece3.388 (PMC3568841; doi:10.1002/ece3.388)

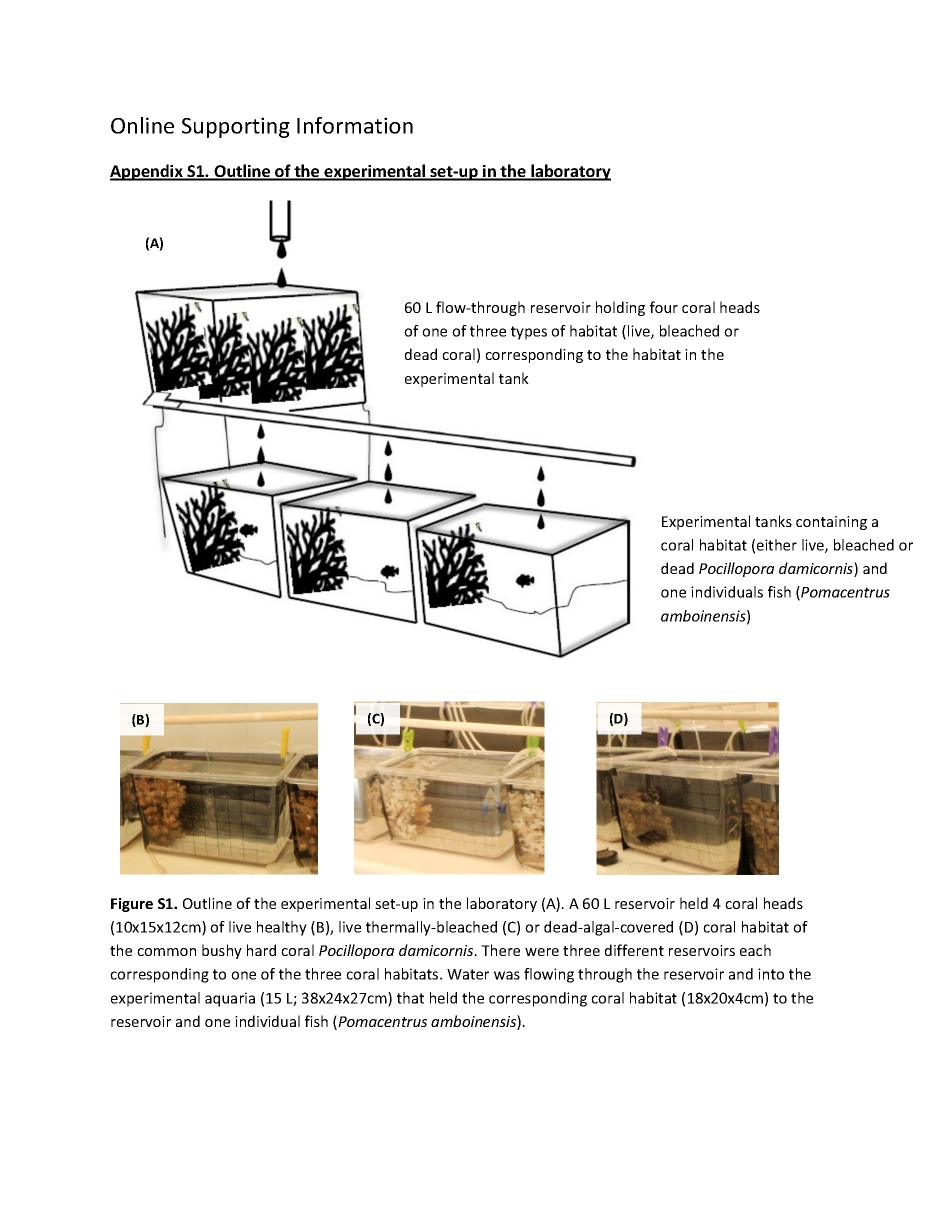

Supplement: Supplementary file 2 [file ece30003-0038-SD2.png]
